# Family environmental risk factors for developmental speech delay in children in Northern China

**DOI:** 10.1038/s41598-021-83554-w

**Published:** 2021-02-16

**Authors:** Shengfu Fan, Ying Zhang, Jiangbo Qin, Xuan Song, Meiyun Wang, Jiangping Ma

**Affiliations:** 1 Department of Foreign Languages, Changzhi Medical College, Changzhi, 046000 China; 2 Deparment of Nursing, Changzhi Medical College, Changzhi, 046000 China; 3 Department of Pediatrics, Heji Hospital Affiliated to Changzhi Medical College, Changzhi, 046000 China; 4 Department of Otolaryngology, Heping Hospital Affiliated to Changzhi Medical College, Changzhi, 046000 China; 5 Emergency Department, Dongying Honggang Hospital, Dongying, 257000 China; 6 Child Healthcare Department, Qilu Children’s Hospital of Shandong University, Ji’nan, 250022 China

**Keywords:** Diseases, Medical research, Risk factors

## Abstract

Most reported risk factors for developmental speech delay (DSD) remain controversial, and studies on paternal influencing factors are rare. This study investigated family environmental risk factors for DSD in northern China. The medical records of 276 patients diagnosed with DSD at four centres between October 2018 and October 2019 were retrospectively analysed. A questionnaire was designed that contained items such as maternal age at the child’s birth, child sex, child age, birth order, family type and parental personality. Patients whose medical records lacked complete information for this investigation were contacted by e-mail or phone. Additionally, 339 families whose children received routine physical examinations at the four involved centres completed the survey. Data were collected, and potential risk factors were analysed using the *t* test or chi-square test; the obtained outcomes were subjected to multivariable logistic regression for further analysis. The multivariable regression showed that older maternal age at the child’s birth (OR = 1.312 (1.192–1.444), P < 0.001), introverted paternal personality (OR = 0.023 (0.011–0.048), P < 0.001), low average parental education level (OR = 2.771 (1.226–6.263), P = 0.014), low monthly family income (OR = 4.447 (1.934–10.222), P < 0.001), and rare parent–child communication (OR = 6.445 (3.441–12.072), P < 0.001) were independent risk factors for DSD in children in North China. The study results may provide useful data for broadening and deepening the understanding of family risk factors for DSD.

## Introduction

Speech delay (SD) in early childhood refers to a condition in which a preschool child develops speech at a noticeably slower rate than other children of the same sex and a similar age^1–3^. Speech and language development delay is a common speech and language disorder in preschool children, with an estimated prevalence of 5%-12% among children aged 2–5 years in the USA^4^ and over 3% among children aged 3 years in China^5^.

SD in children can be caused by a variety of factors, such as visual impairment, hearing impairment, congenital cleft palate, central nervous system impairment and mental disability^1^. However, SD may also occur without noticeable aetiology^6^. Under such a condition, the delay is termed developmental SD (DSD; also known as “specific” speech impairment). The best feature of DSD is that it is “language-specific”; that is, patients with DSD do not display obvious hearing, intelligence, or emotional or cognitive development delays. DSD suppresses the development of young children’s communication ability and may also result in adverse effects on their behavioural and psychological development in the future^5,7^. In the meantime, DSD may affect the emotional status of the parents, which negatively influences the quality of family life^8^.

As DSD children present with no organic defects and few noticeable indicators in early childhood^9^ and as the condition can be greatly improved or even reversed with age^10^, DSD is the type of SD that is most likely to be ignored by parents; it has also received the least attention from scholars and researchers^1^. To prevent the occurrence of DSD, one important task is to identify DSD-associated risk factors^11^, as their identification may increase the possibility for DSD to be prevented, controlled or treated^12^. To date, a few studies have been conducted to identify the risk factors for DSD. Among various potential risk factors, the most frequently reported include positive family history^1^, male sex, low birth weight and preterm birth^8^. In addition, large family size, low parental education level and low family income have also been reported as risk factors for this condition^13,14^. However, there is extensive controversy regarding these reported risk factors. Male sex, caesarean birth and positive family history were found not to be independent risk factors for DSD^15^. Scholars also hold different views on the contributions of maternal and paternal educational levels, childhood illnesses, birth order, and family size to the development of DSD^13^. Therefore, systemic studies on potential risk factors for DSD remain to be conducted. In addition, most of the reported studies have focused on the roles of the mother in the development of DSD, whereas those integrating the contribution of the father to DSD are rare.

Based on the aforementioned information, we conducted the current multicentre retrospective study and investigated the risk factors for DSD based on the medical records of DSD patients and a self-designed questionnaire. To reduce the burden of questionnaire completion as well as possible interference from confounding factors, we focused mainly on familial environmental factors. The results of this study may contribute to broadening and deepening the understanding of family risk factors for DSD.

## Results

### Recruitment

In the patient group, 276 were included, with 22 excluded due to premature birth, 16 excluded due to low birth weight, 7 excluded due to autism, 7 excluded due to a family history of DSD, 36 excluded due to an inability to be contacted, 16 excluded due to being in a single-parent family or a family with parental separation and 12 excluded due to refusing to participate. In the control group, 339 were included, and 11 patients were excluded for providing contradictory information.

According to the data collected, in most families (91%), the mother and father had the same education level. Therefore, we merged the original separate maternal and paternal education levels into the average education level of the family.

The patient group consisted of 173 males (63%) and 103 females (37%), and this sex ratio was significantly different than that of the control group (158 males (47%) and 181 females (53%); P < 0.001). No significant difference in age was observed between the two groups (3.08 ± 1.03 vs. 3.13 ± 1.12; P = 0.784).

### Potential risk factors

The potential influencing family environmental factors for DSD are summarized in Table 1.

**Table 1:** Univariable analysis of the potential familial risk factors for developmental speech delay in children.

Item	Patient (n = 276)	Control (n = 339)	*X*^*2*^	P value
**Maternal age at child’s birth, years**	28.62 ± 4.35	26.50 ± 2.14	N/A	0.044
**Child sex, n/%**			15.815	< 0.001
Male	173 (63%)	158 (47%)		
Female	103 (37%)	181 (53%)		
**Child age, years**	3.08 ± 1.03	3.13 ± 1.12	N/A	0.784
**Birth order, n/%**			7.180	0.007
First	128 (46%)	194 (57%)		
Second or later	148 (54%)	145 (43%)		
**Family type, n/%**			0.009	0.924
Nuclear	265 (96%)	326 (96%)		
Extended	11 (4%)	13 (4%)		
Step	–	–		
**Parental personality, n/%**				
*Maternal*			75.321	< 0.001
Extraverted	26 (9%)	60 (18%)		
Neutral	96 (35%)	205 (60%)		
Introverted	154 (56%)	74 (22%)		
*Paternal*			13.795	0.001
Extraverted	83 (30%)	89 (26%)		
Neutral	104 (38%)	176 (52%)		
Introverted	89 (32%)	74 (22%)		
**Average education level, n/%**			67.252	< 0.001
College or above	58 (21%)	181 (53%)		
High school	197 (71%)	141 (42%)		
Lower than high school	21 (8%)	17 (5%)		
**Family income/month, n/%**			100.380	< 0.001
> 20,000 RMB	15 (5%)	68 (17%)		
10,000–20,000 RMB	103 (37%)	210 (62%)		
5000–10,000 RMB	115 (42%)	61 (18%)		
< 5000 RMB	43 (16%)	10 (3%)		
**Work status, n/%**				
*Maternal*			0.331	0.565
Housewife	73 (27%)	86 (25%)		
Working	193 (73%)	253 (75%)		
*Paternal*			0.154	0.695
Non-working	15 (5%)	16 (5%)		
Working	262 (95%)	323 (95%)		
**Parent–child communication, n/%**			142.331	< 0.001
Very frequent	60 (14%)	178 (53%)		
Frequent	102 (41%)	148 (44%)		
Sometimes or rare	114 (45%)	13 (3%)		
**Child-rearing behaviours, n/%**			6.980	0.031
Rude	–	–		
Strict	145 (53%)	142 (42%)		
Gentle/friendly	99 (36%)	151 (45%)		
Permissive	32 (11%)	46 (13%)		

N/A, not applicable because the *t* test was performed. – means none.

Maternal age at the child’s birth in the patient group was 28.62 ± 4.35 years, which was significantly different from that of the control group (26.50 ± 2.14 years; P = 0.044).

In the patient group, 128 (46%) children were the first child of the family, and 148 (54%) were the second child or later, whereas in the control group, 194 (57%) children were the first child of the family, and 235 (43%) were the second child or later, with a significant difference between the two groups (*x*^2^ = 7.180, P = 0.007).

The patient group was significantly different from the control group in terms of both maternal personality and paternal personality according to chi-square tests (*x*^2^ = 75.321, P < 0.001; *x*^2^ = 13.795, P = 0.001).

The patient group had a significantly lower average education level and lower monthly family income (*x*^2^ = 67.252, P < 0.001 and *x*^2^ = 100.380, P < 0.001, respectively).

The patient group was also significantly different from the control group in terms of parents-child communication frequency and parental child-rearing behaviours (*x*^2^ = 142.331, P < 0.001 and *x*^2^ = 6.980, P = 0.031, respectively).

No significant differences in family type or parental working status were observed between the patient group and the control group (P > 0.05).

### Multivariable logistic regression

The potential risk factors with significant differences between the patient group and the control group according to the *t* test and chi-square test were then subjected to binary logistic regression for further analysis, and the results are summarized in Table 2.

**Table 2:** Independent familial risk factors for developmental speech delay in children according to the multivariable logistic regression analysis.

Item	Odd ratio (95% CI)	P value
Maternal age at child’s birth	1.312 (1.192–1.444)	< 0.001
Paternal personality	0.023 (0.011–0.048)	< 0.001
Average education level	2.771 (1.226–6.263)	0.014
Monthly family income	4.447 (1.934–10.222)	< 0.001
Parent–child communication	6.445 (3.441–12.072)	< 0.001

The independent risk factors for DSD included older maternal age at the child’s birth (OR = 1.312 (1.192–1.444), P < 0.001), introverted parental personality (OR = 0.023 (0.011–0.048), P < 0.001), low average education level (OR = 2.771 (1.226–6.263), P = 0.014), low monthly family income (OR = 4.447 (1.934–10.222), P < 0.001), and rare parent–child communication (OR = 6.445 (3.441–12.072), P < 0.001).

## Discussion

DSD is the consequence of the joint action of a variety of factors, which can be grouped into three general categories, i.e., child factors, parent factors and family and community factors^9^. To date, however, there is great controversy regarding the risk factors of DSD in early childhood^8^. We conducted this multicentre case–control study to investigate the independent family risk factors for this condition, and paternal elements were taken into account.

According to the univariable analyses in this study, the patient group and the control group showed significant differences in maternal age at the child’s birth, birth order, child sex, maternal personality, paternal personality, parental average education level, monthly family income, parent–child communication frequency and child-rearing behaviours. These factors were then subjected to binary logistic regression for further analysis. The multivariable analysis showed that older maternal age at the child’s birth, introverted paternal personality, low average parental education level, low monthly family income and rare parent–child communication were independent risk factors for DSD in children. Although we did not analyse maternal age at the child’s birth in the logistic regression analysis due to the small difference in age between the patient group and the control group, our findings suggest that older maternal age is associated with a greater risk of DSD in children, which was consistent with the literature^13,14^.

Currently, most of the reported independent risk factors for DSD in children remain controversial^13^. Scholars have reported male sex^12,16–18^, birth order^19^ and family type^16^ to be predictor variables of DSD. In the study conducted by Özdas et al.^16^, 105 children with SDs were included for investigation, and the researchers concluded that male sex, birth order and family type were all risk factors for SDs in children. However, in their study, they did not perform multivariable regression analysis to exclude potential confounding factors. The multivariable regression analysis in this study did not support these factors as independent risk factors for DSD. The results of this study were consistent with those reported by Zhu^15^. In addition, our study showed that low average parental education level, low monthly family income and rare parent–child communication were independent risk factors for DSD in children. These results were in line with those reported by Choudhury and Benasich in terms of the education level of the parents^20^, by Singer et al.^21^, by Vernon-Feagans and Cox^22^, and by Zhu^15^.

The strongest feature of the self-designed questionnaire in this study is that we integrated more paternal elements into it. Most of the reported studies on risk factors for DSD have focused either on maternal contributions or on the roles of parents as a whole, whereas those separately integrating paternal elements into investigation have rarely been reported. Moreover, among the small number of published studies that included paternal elements, all focused on the role of parental education level in the development of DSD in children^19,23,24^, and to the best of our knowledge, studies on the associations of other paternal elements, such as paternal personality and work status, with DSD have not been reported. Surprisingly, the multivariable regression analysis in this study showed that introverted paternal personality, rather than maternal personality, was an independent risk factor for DSD. One possible reason for this result is that maternal personality was excluded as a confounding factor of parent–child communication frequency in the multivariable analysis, as it is the mother who normally takes the primary child-caring role in China. Another possible reason is that the sample size of this study was small, which failed to tell the whole story behind the result. To clarify this confusion, large-scale single-parent cohort studies need to be carried out in the future.

This study has some limitations. First, the inclusion time period for the patient group and that for the control group were not matched due to the retrospective nature of this study. To overcome this drawback, prospective studies can be conducted in the future. Second, this study suffered from a small sample size despite being a multi-centre study, and therefore, studies with larger sample sizes should be carried out to verify the outcomes of this study. Third, to reduce the burden of questionnaire completion, some of the items, such as those on personality, the frequency of parent–child communication and child-rearing attitudes, relied on the guardians’ self-assessments, which may have introduced biases to the final outcomes of this study. To overcome this drawback, studies that use a measurement scale for each of these items should be conducted in the future.

In conclusion, older maternal age at the child’s birth, introverted paternal personality, low average parental education level, low monthly family income and rare parent–child communication are independent risk factors for DSD in children in North China. The results of this study may broaden and deepen the understanding of family risk factors for DSD.

## Material and methods

### Patients

Between October 2018 and October 2019, a total of 392 children who were diagnosed with DSD at Heji Hospital affiliated with Changzhi Medical College, Heping Hospital, affiliated with Changzhi Medical College, Shengli Oil Field Central Hospital and Qilu Children’s Hospital of Shandong University, were consecutively included for consideration. These four centres are all located in North China. The medical records of these patients were retrospectively analysed. Among the children, 213 were males, and 179 were females. Their average age was 2.95 ± 1.42 years, and their ages ranged from 2 to 5 years. The diagnostic criteria were based on the Sign-Significant Relations (S–S)-based Language Development Delay Examination Scale (China Rehabilitation Research Center (CRRC) version)^25^. All patients were confirmed to have no visual impairments, hearing impairments, cleft palate, central nervous system damage or mental disability. Patients who met one or more of the following criteria were excluded from the current study: (1) premature birth; (2) low birth weight (< 2.5 kg); (3) other pathological conditions that lead to speech disorders, such as autism; (4) a family history of DSD; 5) incomplete medical records and absence of contact information; (6) single-parent family or parental separation; or (7) refusal to participate.

In addition, healthy children who underwent routine health examinations at these centres between August and December 2019 were consecutively included for consideration. Before the questionnaire distribution, the guardians of the children were briefly questioned, and those meeting any of the exclusion criteria (the same for the patient group) were excluded from further investigation. A total of 350 questionnaires were administered, and the included children constituted the potential control group.

**Figure 1:**
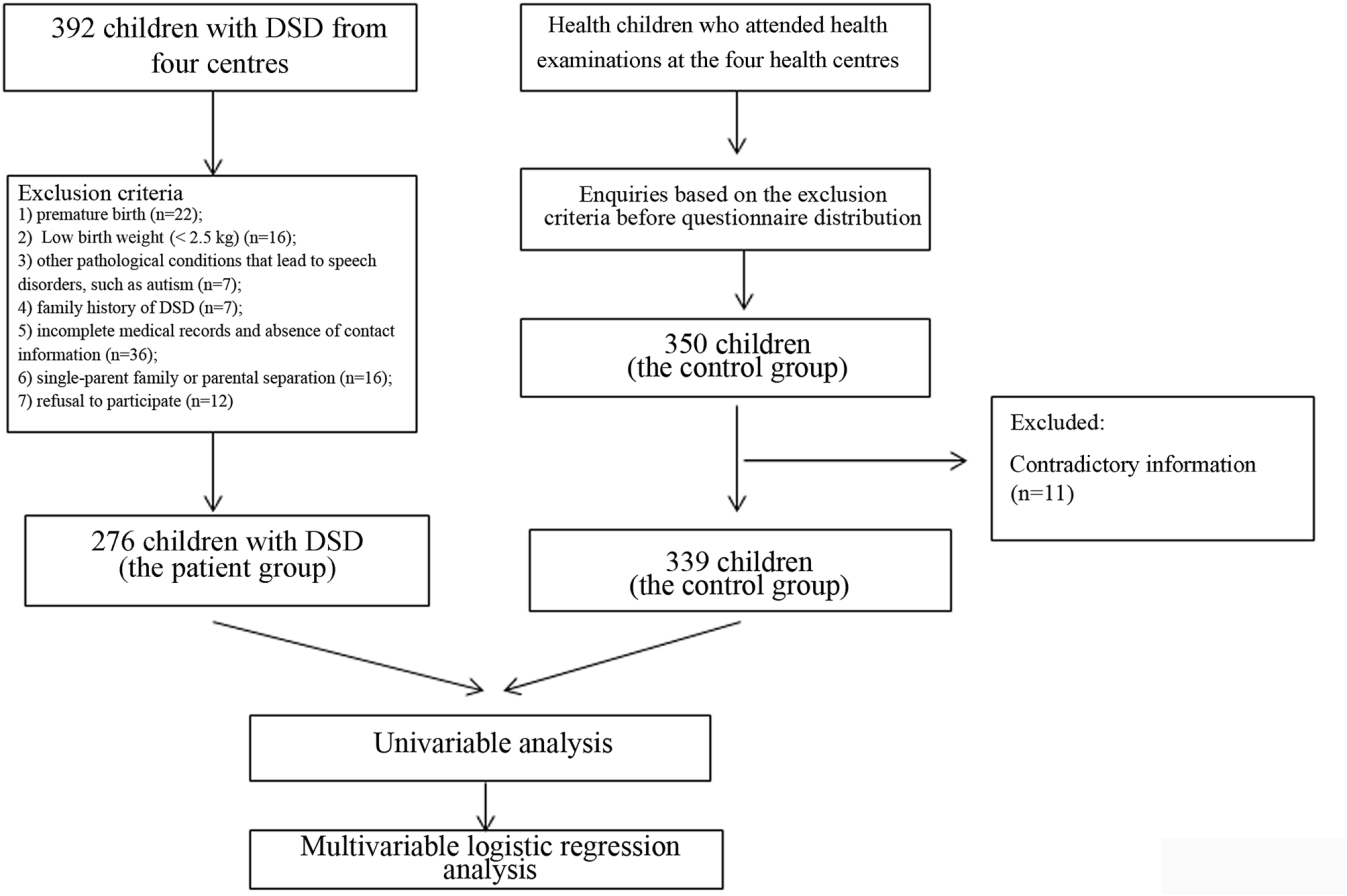
Fluxogram of participant recruitment in this study.

A fluxogram of the recruitment process of this study is shown in Fig. 1.

### Questionnaire

The investigation was performed with a self-designed questionnaire. Part of the content of the questionnaire was designed based on the literature^15,16^, including the items on maternal age at the child’s birth, birth order, child sex, family type, maternal personality, monthly family income, and child-rearing behaviours, and each of these items contained two- or three-point response scales. The difference between this questionnaire form and those in the literature lay in the integration of paternal elements, which included paternal personality, paternal education level and paternal work status (S1 File).

### Data collection

#### Patient group

Because some of the medical records were not completely consistent with the questionnaire in content, we contacted the patients’ guardians via the contact information they had provided (phone number or email address). The guardians were informed of the aim of the survey, and data confidentiality was clarified. Those who were reluctant or refused to participate or who were unable to be contacted and lacked complete medical records were excluded.

#### Control group

Between August and December 2019, we distributed questionnaires to the guardians of the healthy children who underwent routine physical examination at the involved centres. The selected children were aged from 2 to 5 years. They did not have complaints of DSD and were medically confirmed to be without developmental abnormalities. Before the questionnaire completion, the guardians were informed of the aim of this survey. In the case of the absence of one patient, the questionnaire was allowed to be taken home and then returned to the centre after completion at the appointed time.

#### Data collection

Data were collected by two investigators. When contradictory content was observed in a questionnaire, the inclusion or exclusion of the involved family was determined through discussion between the two investigators. If disagreement remained, a third investigator participated in the final decision making.

## Ethical statements

The procedures of this study were in accordance with the Declaration of Helsinki and approved by the Ethics Committee of Heji Hospital affiliated with Changzhi Medical College (approval no., 201,907). Written informed consent to the research purposes was obtained from the parents of the children.

### Statistical analysis

Data were processed with SPSS 24.0. Measurement data are presented as the mean ± standard deviation (M ± SD), and count data are presented as the number of cases and percentage. The independent *t* test or the chi-square test was used to compare the two groups. Multivariable step-forward logistic regression analysis was performed to exclude potential confounding factors. P < 0.05 was considered to be statistically significant.

## Supplementary Information

Supplementary Information.

## Data Availability

All data used for analysis in this study can be found within the article and supplementary file.
