# Supplementary material for: Family environmental risk factors for developmental speech delay in children in Northern China
Source: Sci Rep. 2021 Feb 16;11:3924. doi: 10.1038/s41598-021-83554-w (PMC7887192; doi:10.1038/s41598-021-83554-w)
Supplement: Supplementary file 1 — Supplementary Information. [file 41598_2021_83554_MOESM1_ESM.doc]

1. Maternal age at the child’s birth _________________ (years)

2. Child age ___________________ (years)

3. Child sex Male ( ) Female ( )

4. Birth order First ( ) Second or later ( )

5. Family type

Nuclear ( ) Extended ( ) Step ( )

6. Parental personality

Mother

Extraverted ( ) Neutral ( ) Introverted ( )

Father

Extraverted ( ) Neutral ( ) Introverted ( )

7. Education level

Mother

College or above ( )

High school ( )

Lower than high school ( )

Father

College or above ( )

High school ( )

Lower than high school ( )

8. Family income/month

>20000 RMB ( )

10000-20000 RMB ( )

5000-10000 RMB ( )

<5000 RMB ( )

9. Work status

Mother Housewife ( ) Working ( )

Father Non-working ( ) Working ( )

10. Parent-child communication

Very frequency ( ) Frequency ( ) Sometimes or rare ( )

11. Child rearing manner

Rude ( ) Strict ( ) Gentle/friendly ( ) Spoiling ( )
